# Determinants of postnatal care service utilization among married women in rural areas in western Ethiopia

**DOI:** 10.1186/s41043-022-00320-y

**Published:** 2022-08-19

**Authors:** Tesfalidet Beyene, Alemu Sufa Melka, Birhanu Yadecha

**Affiliations:** 1 College of Medical and Health Sciences, Wollega University, Nekemte, Oromia Ethiopia; 2 University of Newcastle, Newcastle, NSW Australia

**Keywords:** Postnatal care, Utilization, Rural, Women, Ethiopia

## Abstract

**Background:**

Worldwide studies have shown that three-fourths of the total deaths during the neonatal period occur in the first week of the postnatal period. However, most of these deaths can be prevented with care during pregnancy, childbirth, and postnatal care. According to the 2016 Ethiopia Demographic and Health Survey report, 17% of women in Ethiopia had received postnatal care after childbirth. This study aimed to identify determinants of postnatal care service utilization among married women in rural areas in Western Ethiopia.

**Methods:**

A community-based cross-sectional study was conducted among 798 women who had given birth in the past 2 years prior to the survey between 2 and 31 January 2015. A pre-tested structured questionnaire was used to collect the data. Multivariable logistic regression was employed to determine factors affecting utilization of postnatal care. Adjusted odds ratios (AOR) and 95% confidence intervals (CI) were used to assess the strength of the associations.

**Results:**

The study showed that 188 (23.6%) women utilized postnatal care services during their last pregnancy. Women’s educational level (AOR = 3.29, 95%CI = 1.89–5.73), utilization of antenatal care (AOR = 2.07, 95%CI = 1.28–3.36), awareness on the advantage of postnatal care (AOR = 2.10, 95%CI = 1.41–3.13), and knowledge of at least one danger sign during the postnatal period (AOR = 3.04, 95%CI = 2.07–4.46) showed a significant positive association with the utilization of postnatal care.

**Conclusion:**

Educating women and creating awareness of maternal health care services during pregnancy increase the utilization of postnatal care services. Health care professionals should provide information on the importance of postnatal care for pregnant women during antenatal care visits.

## Introduction

Mothers, newborns, and children are closely linked in life and health care needs. In the past, maternal and child health care policies and programs addressed the health of the mother and child separately, resulting in gaps in care that affect newborn babies. Any health problem that affects the health of the mother during pregnancy, childbirth, and the postnatal period affects the newborns as well [1].

According to the World Health Organization (WHO), every day about 830 women die due to health problems related to pregnancy or childbirth. Nearly, all of these deaths occur in a low-resource country where a large number of births do not take place at health institutions and where postnatal care (PNC) is either not accessible or is of poor quality. However, most incidents can be prevented with care during pregnancy, childbirth, and postnatal care. Sub-Saharan African countries accounted for more than half of maternal and neonatal death. Most of these deaths occur during childbirth and the first week of the postnatal period [2–7]. Studies have shown that three-fourths of the total deaths during the neonatal period occur in the first week of the postnatal period [6, 7].

A stud done in 2005 has demonstrated that if routine PNC service and curative care after childbirth reach 90% of newborns and their mothers, 10 to 27% of newborn deaths can be prevented. In other words, high PNC coverage could save up to 310,000 newborn lives a year [8]. Furthermore, in 2012, a meta-analysis showed that receiving care from antenatal to postnatal periods may reduce the risk of neonatal, perinatal, and maternal mortality by 15% [9]. The use of quality and evidence-based PNC is an effective approach to reduce maternal and newborn morbidity and mortality, particularly in places where the health status of women is poor and affected. To maintain the health status of women and the newborn, every woman should have a postnatal checkup after delivery [10–12]. The rates of the provision of skilled care are lower during the postnatal period when compared to rates before and during childbirth [7]. WHO stated that the postnatal period begins immediately after the birth of the baby and extends up to 6 weeks after birth. In this period, major physiological and psychological changes occur which determines the well-being of mothers and newborns [7, 13].

The main objectives of PNC services are to identify, promote and maintain the health of the woman and the newborn. It also enables health care providers to identify postnatal problems, that may include potential complications, and to provide prompt treatment [13, 14]. In low-income countries, less attention was given to postnatal care, although most maternal and newborn deaths occurred within the first week of the postnatal period. Across Sub-Saharan countries, many mothers are not receiving the PNC appropriately [15–18]. According to the Ethiopia Demographic and Health Survey (EDHS) report, PNC service utilization is extremely low—only 17% of women received PNC within the recommended time [19].

There is a strong rationale for improving the utilization of PNC services to improve maternal and newborn health. For this study, we used data from previously published work in BMC Research Notes and the aim of the previously published study was on the use of institutional delivery service [20]. To the best of our knowledge, no study identifies factors affecting the uptake of PNC service among mothers in rural areas in Western Ethiopia. The finding of this study will help policymakers and program implementers to identify factors associated with the utilization of PNC services and develop strategies to tackle the problem of underutilization of PNC services.

## Methods

### Study design and setting

This was a population-based cross-sectional study involving mothers who gave birth in the last 2 years prior to the survey. The study was conducted between 2 and 31 January 2015 in rural areas of East Wollega Zone, western Ethiopia. East Wollega Zone is located in Oromia Regional state with a population of 1,230,402 among which 49.9% are males and 50.1% are females. The majority of the population (86%) live in rural areas. Nekemte is the capital city of the zone which is located 331 km West of Addis Ababa with a population of 76,817 [21]. The study participants were randomly selected married women in the reproductive age groups who gave at least one birth in the last 2 years preceding the survey. Women who were ill and unable to provide informed consent were excluded from the study.

### Sample size and sampling procedure

A sample size of 801 was determined using single population proportion based on the assumptions of the magnitude of PNC service utilization in Hossaina 51.4% (*p* = 0.514) [22], a 5% marginal error, a 95% level of confidence, a design effect of 2 and 5% of non-response rate.

A multi-stage sampling procedure was done to select study participants. From eighteen districts found in the East Wollega Zone, a total of ten rural lower administrative levels (Kebeles) were randomly selected. The first phase of the data collection included listing of houses and household members to identify all women who had delivered in the last 2 years preceding the survey. In the second phase, the calculated sample size was proportionally allocated to each lower administrative level based on the number of married women who gave birth in the last 2 years. In the third phase, households were randomly selected from each lower administrative level to allocate the sample size. Finally, eligible study subjects were interviewed from each selected household.

### Data collection procedures

Data were collected through face-to-face interviews using a structured and pre-tested questionnaire, which is adapted and modified from different works of the literature [19, 22–24]. The questionnaire was prepared in English and then translated into the local language of Afan Oromo.

The data were collected by female interviewers and checked by supervisors. Three days of training were given for data collectors and supervisors which included the objective of the study, consent process, techniques of structured interviewing, and specific administration of each item in the questionnaire. All completed questionnaires were revised each night and issues that arose during data collection were addressed in morning sessions with supervisors.

### Data analysis

Data were entered into Epi-Info version 6.5 software and exported to the SPSS version 20.0 for analysis. The data were summarized by frequency tables and summary statistics. Bivariate analysis was conducted to determine the association between PNC service utilization and independent variables (socio-demographic characteristics, reproductive characteristics and awareness and utilization of maternal health care services). Furthermore, multivariable analysis was performed to control the effect of confounders. Adjusted odds ratio (AOR) and confidence intervals (CI) were used to determine the strength of the association. A *p* value less than 0.05 considered statistically significant. The bar chart was prepared using Graph prism.

## Results

### Socio-demographic characteristics of the respondents

A total of seven hundred ninety-eight mothers participated in the study which gives a response rate of 99.5%. Most of the respondents (64.0%) were in the age group of 25–34 years, with a mean age of 29.5 years. The majority (94.2%) of the respondents identified themselves ethnically as Oromo and 58.0% identified as protestant. Nearly, half (47.6%) of respondents could not read or write. Four in five (79.6%) respondents were housewives, and 81% of their husbands were farmers. Respondents had a mean monthly income of 841.01 ETB and 45.0% had radio and/or TV (Table 1).

**Table 1:** Socio-demographic characteristics of respondents who gave birth in the last 2 years in rural areas in western Ethiopia, January 2015

Variables (798)	Number (%)
*Age category*	
15–24	116(14.5)
25–34	511(64.0)
35–44	171(21.4)
*Ethnicity*	
Oromo	752(94.2)
Amhara	40(5.0)
Tigre	6(0.8)
*Religion*	
Protestant	463(58.0)
Ethiopian orthodox	312(39.1)
Catholic	3(0.4)
Muslim	19(2.4)
Others*	1(0.1)
*Educational status of the respondent*	
Can’t read and write	380(47.6)
Can read and write	57(7.1)
Grade 1–4	154(19.3)
Grade 5–8	78(9.8)
Secondary	117(14.7)
College and above	12(1.5)
*Educational status of the husband*	
Can’t read and write	88(11.0)
Can read and write	132(16.5)
Grade 1–4	232(29.1)
Grade 5–8	196(24.6)
Secondary	114(14.3)
College and above	36(4.5)
*Occupational status of the respondents*	
Housewife	635 (79.6)
Governmental employee	37(4.6)
Daily laborer	77(9.6)
Merchant	40(5.0)
Student	9 (1.1)
*Occupational status of the husband*	
Farmer	647(81.1)
Governmental employee	71(8.9)
Daily laborer	29(3.6)
Merchant	35(4.4)
Student	16(2.0)
*Income (ETB)*	
< 490	200(25.1)
491–700	216(27.1)
701–1000	230(28.8)
> 1000	152(19.0)
Mean	841.01ETB
*Have radio/TV*	
Yes	359(45.0)
No	439(55.0)

Other* = Wakefeta, Jehovah’s Witness ETB (Ethiopian Birr) 1$US = 22.17ETB

### Reproductive characteristics of the respondents

Most (87.3%) of the respondents had more than one child. The majority (92.1%) of respondents had no history of abortion and 60.0% were aware of the danger signs of pregnancy (Table 2).

**Table 2:** Reproductive characteristics of respondents who gave birth in the last 2 years in rural areas in western Ethiopia, January 2015

Variables	Number (%)
*Parity (798)*	
1	101(12.7)
2–4	507(63.5)
≥ 5	190(23.8)
*Abortion in lifetime*	
Yes	63(7.9)
No	735(92.1)
*Knew danger sign of pregnancy*	
Yes	479(60.0)
No	319(40.0)

### Awareness and utilization of maternal health care service

Two hundred ninety-nine (37.5%) respondents faced problems during pregnancy, and 61.9% had attended ANC appointments at least once. Over four in seven (60.3%) respondents gave birth at home. Most (86.6%) respondents had heard about PNC, 23.6% utilized PNC services, and 54.1% knew the advantage of PNC services. The major source of information regarding PNC was health extension workers (82.2%) (Table 3).

**Table 3:** Awareness and utilization of maternal health care service of the respondents who gave birth in the last 2 years in rural areas in western Ethiopia, January 2015

Variables (798)	Number (%)
*Had a problem during last pregnancy*	
Yes	299(37.5)
No	499(62.5)
*Mention problem during last pregnancy*	
Vaginal bleeding	29(9.7)
Severe headache	111(37.1)
Severe abdominal pain	39(13.0)
Drowsiness	102(34.1)
Facia swelling	24(8.0)
Hand swelling	3(1.0)
Persistent vomiting	175(58.5)
*Antenatal care service utilization*	
Yes	494(61.9)
No	304(38.1%)
*Place of delivery*	
Home	481(60.3)
Health facility	317(39.7)
*Heard about PNC service*	
Yes	691(86.6)
No	107(13.4)
*Source of information about PNC*	
Health institution	287(41.5)
Radio or Tv	99(14.3)
Health extension worker	568(82.2)
Family members or relatives	35(5.1)
Others	8(1.6)
*Know the advantage of PNC service*	
Yes	374(54.1)
No	317(45.9)
*Mentioned advantages*	
To detect the health problem after delivery	156(41.7)
Infant feeding	182(48.7)
Give opportunity to get family planning	226(60.4)
Check the condition of the newborn	157(39.3)
*Postnatal care service utilization*	
Yes	188(23.6)
No	610(76.4)

### Main reasons for utilization of postnatal care

**Fig. 1:**
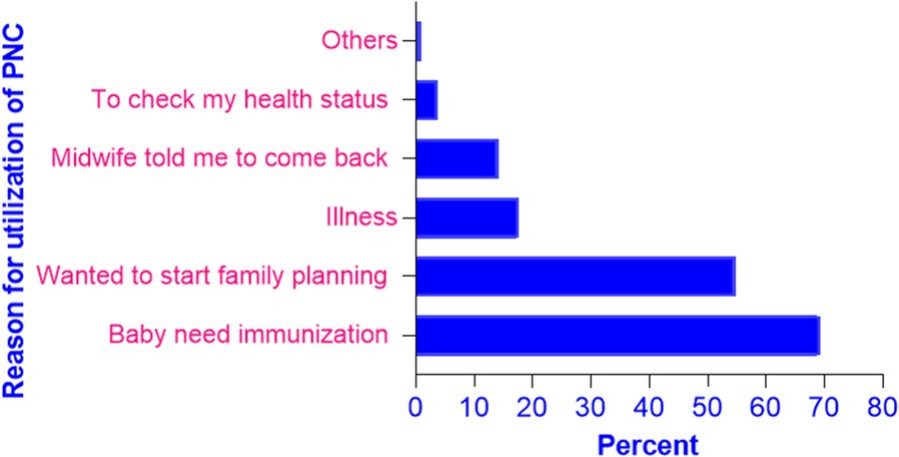
Bar graphs showing reasons for utilization of PNC among women who gave birth in the last 2 years in rural areas in western Ethiopia, January 2015

The major reasons for attending postnatal care were immunization of the newborns (69.2%), followed by wanting to start family planning (54.6%) (Fig. 1).

### Predictors of postnatal care service utilization

Multivariable analysis showed that respondents who finished secondary and post-secondary school education were three times more likely to utilize PNC services when compared to those who had primary school education and below (AOR = 3.29, 95% CI 1.89–5.73). Those respondents who utilized ANC in the last pregnancy were two times more likely to seek PNC than their counterparts (AOR = 2.07, 95% CI 1.28–3.36). Respondents who knew the advantage of PNC were also twice more likely to receive PNC than those who were not aware of the advantages (AOR = 2.10, 95% CI 1.41–3.13). Respondents who knew at least one health problem occurring during the postnatal period were three times more likely to use PNC than those who did not have the knowledge (AOR = 3.04, 95% CI 2.07–4.46) (Table 4).

**Table 4:** A multivariable logistic regression on predictors of postnatal care service use in rural areas in western Ethiopia, January 2015

Characteristics	Utilization of postnatal care		Crude OR	Adjusted OR
	Yes (%)	No (%)	OR(CI)	OR(CI)
*Age category in years*				
15–24	35(30.2)	81(69.8)	2.11(1.21–3.71)	1.32(0.57–3.04)
25–34	124(24.3)	387(75.7)	1.57(1.00–2.46)	0.90(0.49–1.67)
35–44	29(17.0)	142(83.0)	1	1
*Education of respondents*				
Below and primary	122(18.2)	547(81.8)	1	1
Secondary and above	66(51.2)	63(48.8)	4.70(3.16–6.99)	3.29(1.89–5.73)*
*Education of husband*				
Below and primary	133(20.5)	515(79.5)	1	1
Secondary and above	55(36.7)	95(63.3)	2.24(1.53–3.29)	0.93(0.57–1.54)
*Occupation of respondents*				
Housewife	137(21.6)	498(78.4)	1	1
Others	51(31.3)	112(68.7)	1.66(1.13–2.42)	1.10(0.67–1.80)*
*Utilization of ANC in the last pregnancy*				
Yes	147(29.8)	347(70.2)	2.72(1.86–3.98)	2.07(1.28–3.36)*
No	41(13.5)	263 (86.5)	1	1
*Last delivery*				
Health facility	90(28.4)	227(71.6)	1.55(1.11–2.16)	0.96(0.64–1.44)
Home	98(20.4)	383(79.6)	1	1
*Parity*				
1	25(24.8)	76(75.2)	1.51(0.84–2.71)	0.45(0.19–1.06)
2–4	129(25.4)	378(74.6)	1.57(1.03–2.39)	1.09(0.64–1.87)
≥ 5	34(17.9)	156(82.1)	1	1
*Know the advantage of PNC*				
Yes	136(36.4)	238(63.6)	2.91(2.02–4.19)	2.10(1.41–3.13)*
No	52(16.4)	265(83.6)	1	1
*Knew at least one health problem occur during postnatal period*				
Yes	123(41.0)	177(59.0)	3.49(2.45–4.96)	3.04(2.07–4.46)
No	65(16.6)	326(83.4)	1	1
*Possessing radio/and TV*				
Yes	111(30.9)	248(69.1)	2.10(1.51–2.93)	1.25(0.84–1.87)
No	77(17.5)	362(82.5)	1	1

*Statistically significant (*p* value < 0.05); 1: Reference category

## Discussion

In low-income countries, about 40% of women experience complications after childbirth and 15% develop potentially life-threatening complications. When compared with other maternal health care services, the magnitude of PNC tends to be low [25, 26]. The current study has shown that the utilization of PNC service in the study area was 23.6%. The magnitude of PNC is high compared to the national report of Ethiopia and low compared to other individual studies done in Ethiopia [18, 19, 22]. The difference may be because the current study included only rural women who may not have more access to health care services.

Studies have indicated that maternal education has a great role in the utilization of maternal health care services [20, 27–32]. In the present study, women who have at least a secondary education were more likely to receive PNC than those without that level of education. This finding agrees with other studies done elsewhere [28, 30, 31]. The possible reason could be that education helps women gain greater decision-making power and enables them to communicate with their families and relatives to access maternal healthcare services.

One of the important findings of our study involved the effects of ANC use on PNC service use. This study consistent with other studies done in Bangladesh and Nepal [25, 33]. This indicates that women who were attending ANC services might have a better chance of exposure to other maternal healthcare services, counseling, and encouragement. During the ANC visits, the women may receive important information about the health problems that occur during pregnancy, delivery and after childbirth. This increases the awareness of the women about complications and increases the utilization of PNC service.

Use of healthcare services in general is influenced by the awareness of the healthcare seekers [34, 35]. In the current study, women who have an awareness of the advantages of PNC services were more likely to use PNC services. The reason might be women who have awareness on the advantage of the services fear about the complication and the consequence of the health problems. Likewise, women who were familiar with post-partum complications were more likely to use PNC unlike those who did not recognize potential complications following delivery. Previous studies also reported the same findings [18, 22]. The reason might be a perceived threat that could motivate them to attend maternal health care services.

The strength of this study is that it is community-based; it could reflect the real experiences of mothers during the data collection period. This survey report has some potential limitations such as recall bias and not all maternal healthcare determinants are included in the study due to the scope of the study. The cross-sectional nature of the data was also a limitation of this study.

## Conclusions

This study showed that utilization of PNC services was 23.6%. Women’s literacy status; utilization of ANC; awareness of the advantage of PNC; and knowledge of at least one health problem that could occur during the postnatal period were found to be important determinants of PNC service utilization. Empowering women with education and creating awareness on maternal healthcare services during pregnancy increase the utilization of PNC. Health care professionals should provide information on the importance of PNC to pregnant women during ANC visits.

## Data Availability

Data essential for conclusion are included in this manuscript. Additional data can be obtained from the corresponding author on reasonable request.
